# Safety and efficacy of Razumab™ (world’s first biosimilar ranibizumab) in wet age-related macular degeneration: a post-marketing, prospective ASSET study

**DOI:** 10.1186/s40942-021-00293-w

**Published:** 2021-03-24

**Authors:** Shashikant Sharma, Vishali Gupta, Aniruddha Maiti, Sribhargava Natesh, Sandeep Saxena, Vivek Dave, Vimal Parmar, Raju Sampangi, Hemanth Murthy, Sandhya Dharwadkar, Naresh Kumar Yadav, Shrinivas Joshi, Rahul Mayor, Dhanashree Ratra, Soumyava Basu, Neha Goel, Alok Chaturvedi, Ronak Patel, Vinu Jose

**Affiliations:** 1 Medical Affairs, Intas Pharmaceuticals Ltd, Ahmedabad, Gujarat India; 2 Advanced Eye Centre, Postgraduate Institute of Medical Education and Research, Chandigarh, India; 3 Susrut Eye Foundation & Research Centre, Kolkata, West Bengal India; 4 Nethra Eye Hospital, Bangalore, Karnataka India; 5 Department of Ophthalmology, King George’s Medical University, Lucknow, Uttar Pradesh India; 6 L V Prasad Eye Institute, Hyderabad, Telangana India; 7 PBMA’s H. V. Desai Eye Hospital, Pune, Maharashtra India; 8 Gurushree Hi-Tech Multi Speciality Hospital, Bangalore, Karnataka India; 9 Retina Institute of Karnataka, Bangalore, Karnataka India; 10 K.R. Hospital, Mysore Medical College & Research Institute, Mysore, India; 11 Narayana Nethralaya, Bangalore, Karnataka India; 12 M M Joshi Eye Institute, Hubli, Karnataka India; 13 Dr. Shroff’s Charity Eye Hospital, New Delhi, India; 14 Sankara Nethralaya, Chennai, Tamil Nadu India; 15 L V Prasad Eye Institute, Bhubaneswar, Odisha India; 16 ICARE Eye Hospital & Post Graduate Institute, Noida, Uttar Pradesh India; 17 Present Address: Eye7 Chaudhary Eye Centre, New Delhi, India; 18 Department of Biostatistics and Programming, Lambda Therapeutic Research Ltd., Ahmedabad, Gujarat India; 19 Clinical Development & Medical Affairs, Intas Pharmaceuticals Ltd. (Biopharma), Ahmedabad, Gujarat India

**Keywords:** Razumab, Biosimilar ranibizumab, Neovascular, Wet age-related macular degeneration, AMD, Safety

## Abstract

**Background:**

Razumab™ (world’s first biosimilar ranibizumab) is approved for several macular disorders including wet age-related macular degeneration (AMD). We evaluated the safety and efficacy of biosimilar ranibizumab in wet AMD.

**Methods:**

This prospective, multicentre, rAnibizumab bioSimilar Safety Efficacy postmarkeTing (ASSET) study enrolled patients aged ≥ 50 years with wet AMD having best-corrected visual acuity (BCVA) between 20/40 and 20/320. The patients received intravitreal biosimilar ranibizumab 0.5 mg every 4 weeks for 24 weeks. Safety endpoints included the incidence of adverse events (AEs), serious AEs (SAEs), and immunoreactivity after 6 months. The efficacy endpoints were the proportion of patients who lose fewer than 15 letters, increase in BCVA, change in central retinal thickness (CRT), and change in Visual Function Questionnaire-25 (VFQ-25) score, from baseline to 24 weeks.

**Results:**

Of the 126 enrolled patients, majority (95.24%) of the patients received all 6 doses of biosimilar ranibizumab (total 3 mg). Nineteen AEs were reported (n = 16; 12.7%); majority (78.9%) were mild. There were no serious AEs reported, except one AE of death which was unrelated to the study drug. None of the patients discontinued the study due to an AE. The most common ocular AE was increase in intraocular pressure (4 events) and non-ocular AE was pyrexia (5 events). A total of 7.9% (10/126) patients prior to dosing and 7.1% (9/126) patients post-treatment were positive for anti-ranibizumab antibodies. No AEs suggestive of immunogenicity were noted. At 24-weeks, 97.60% patients in the intent-to-treat (ITT) population (N = 125) and 97.41% patients in the per-protocol (PP) population (N = 116) lost < 15 letters from baseline visual acuity. In the ITT and PP populations, 31.20% and 32.76% patients, respectively, showed improved visual acuity by ≥ 15 letters. Significant improvements in BCVA (mean difference: 8.8, 9.2, p < 0.001 for ITT, PP) and VFQ-25 (8.5, 9.2, p < 0.001 for ITT, PP) were seen; CRT reduced significantly (125 µm, 119.3 µm, p < 0.001 for ITT, PP).

**Conclusion:**

Razumab™ (world’s first biosimilar ranibizumab) was well-tolerated without new safety concerns and significantly improved visual acuity in wet AMD patients.

*Trial registration* CTRI/2016/03/006739. Registered 18 March 2016—Prospectively registered, http://ctri.nic.in/Clinicaltrials/pmaindet2.php?trialid=13141&EncHid=&userName=2016/03/006739

**Supplementary Information:**

The online version contains supplementary material available at 10.1186/s40942-021-00293-w.

## Background

Age-related macular degeneration (AMD) is a progressive neurodegenerative disease and a leading cause of visual loss in elderly patients [1]. Wet (exudative) AMD begins with neovascularization from choroid due to defects in the Bruch’s membrane. Neovascular vessels proliferate underneath the retinal pigment epithelium, and leakage from these vessels leads to retinal elevation resulting in blurring of vision [2, 3]. In India, the prevalence of AMD is estimated between 1.4% and 3.1% with advanced age (> 65 years) being the most common factor affecting the prevalence of AMD [4]. The global prevalence of AMD is estimated at ~ 8.7% [5]. Though wet AMD accounts only for 10% to 15% of all the AMD cases [6], 80% of these patients develop severe vision loss [7].

Vascular endothelial growth factor (VEGF)-A plays a vital role for angiogenesis and vascular permeability, and it is overexpressed in wet AMD [8, 9]. Anti-VEGF agents, pegaptanib, ranibizumab and aflibercept are approved [3, 10] while bevacizumab is used off-label for the treatment of wet AMD [11, 12]. Ranibizumab is a humanized monoclonal antibody approved in the United States since June 2006 [13] and in Europe since January 2007 [14]. Ranibizumab binds with a high affinity to VEGF-A isoforms and prevents the interaction of VEGF-A with its receptors VEGFR-1 and VEGFR-2 on the surface of endothelial cells [2].

Intas Pharmaceuticals Limited, India, has developed Razumab™, the world’s first biosimilar ranibizumab, which has been approved (permission no. MF-35/2015 BULK-36/2015, Dated 20^th^ February 2015) by the highest Indian regulatory authority ‘Drugs Controller General of India (DCGI)’ for the treatment of neovascular (wet) AMD, diabetic macular edema (DME), macular edema following retinal vein occlusion (RVO) and for visual impairment due to choroidal neovascularization (CNV) secondary to pathologic myopia [15]. The efficacy and safety of biosimilar ranibizumab has been established with prospective as well as retrospective studies for the treatment of several macular disorders including wet AMD, DME, RVO and myopic CNV [16–22]. In this article, we present the results on the safety, immunogenicity and efficacy of biosimilar ranibizumab in Indian patients with wet AMD from a prospective, post-marketing study.

## Methods

### Study design

The ASSET (rAnibizumab bioSimilar Safety Efficacy postmarkeTing) was a phase 4, prospective, interventional, single-arm, non-comparative, multicentre, postmarketing study to evaluate the safety and efficacy of the world’s first biosimilar ranibizumab, Razumab™, in patients with wet AMD (Clinical Trial Registry Number: CTRI/2016/03/006739). The first patient was enrolled on 01 June 2016 and the last patient completed the study on 06 June 2018. The study was conducted at 16 sites across India. The study protocol was approved by an institutional ethics committee of the respective study center. The study was conducted in accordance with the Declaration of Helsinki, good clinical practices and relevant regulatory guidelines. All patients provided written informed consent before enrolment into the study.

### Patients

We enrolled patients of either gender, aged ≥ 50 years, having active primary or recurrent sub-foveal lesion with CNV secondary to AMD, involving the foveal center, and having a best corrected visual acuity (BCVA) of 20/40 to 20/320 using the Early Treatment Diabetic Retinopathy Study (ETDRS) chart (Snellen equivalent) in the study eye. Only one eye was evaluated, and if both eyes were eligible for inclusion, the eye with the better visual acuity was selected unless the investigator chose the other eye for treatment on medical grounds.

Patients were excluded if they had received prior treatment with any intravitreal drug, ranibizumab, verteporfin or photodynamic therapy within the past three months; or had received prior treatment with systemic or intravitreal bevacizumab within the last six months; or underwent extrafoveal laser photocoagulation within one month in the study eye; had subfoveal fibrosis or atrophy in the study eye, had CNV in either of the two-eyes due to causes other than AMD; had intraocular condition that could require any intervention during the six months study period; had vitreous haemorrhage or rhegmatogenous retinal detachment or macular hole (stage 3 or 4); had active intraocular inflammation or ongoing infection; had a recent history of stroke, or a patient with known hypersensitivity to ranibizumab.

### Treatment and follow-up

**Fig. 1:**
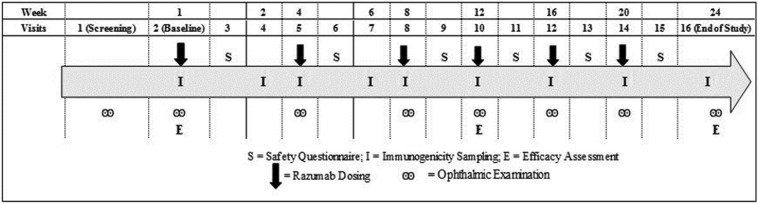
Study assessment schedule

Patients were screened within 4 weeks before study enrolment. Eligible patients received intravitreal injections of biosimilar ranibizumab at a dose of 0.5 mg every 4 weeks for 24 weeks for a total of 6 doses. A total of 16 study visits were planned for each enrolled patient including the screening visit and the end of study visit (Fig. 1). Patients were followed-up for evaluations the next day after study drug administration.

### Safety assessments

The safety endpoints included the incidence of adverse events (AEs) and serious AEs (SAEs) including any hypersensitivity reactions, any significant laboratory abnormalities, and the proportion of patients developing anti-ranibizumab antibody after 6 months of treatment.

Fundus fluorescein angiography (FFA) was performed for all patients on the day of screening to assess the lesion severity. The spectral domain-optical coherence tomography (SD-OCT) examinations were performed on the day of screening, baseline visit and at 3- and 6-months post-treatment visits. Slit-lamp examination, indirect ophthalmoscopy, and intraocular pressure measurements were done at screening, each dose administration visits and at the end of the study. Ophthalmic examination was done for safety assessment on the subsequent day of dosing using the safety questionnaire (Additional file 1: Appendix S1).

Other safety assessments included physical examination, vital signs assessment, chest X-ray, electrocardiograph, and routine laboratory evaluations. Adverse events were monitored during the study and coded using Medical Dictionary for Regulatory Activities (MedDRA) version 20.1.

### Immunogenicity assessments

Immunogenicity was evaluated by assessing the presence of serum anti-ranibizumab antibodies in all patients. A total of 9 samples (baseline, Weeks 2, 4, 6, 8, 12, 16, 20 and 24), each of 6 mL, were collected from each patient to detect anti-ranibizumab antibodies in the serum. Blood samples were collected through the needle from a venipuncture site with the arm in downward position, and collected in vacutainers placed upright in a rack kept in ice cold water bath until centrifugation. The serum samples were centrifuged at 3000 ± 100 rpm for 10 min at room temperature (18–25 °C) to separate the serum. The serum samples were transferred using pasture pipette (dropper) to polypropylene cryovial in two aliquots (around 500 μL in first aliquot and remaining volume in second aliquot) and stored at a temperature of − 22 °C ± 5 °C or below for interim storage until shipment for analysis. Samples were at controlled temperature (− 22 °C ± 5 °C or below)/packed with dry ice for transport along with data logger. After receiving the samples at the Bioanalytical Laboratory of Lambda Therapeutic Research Limited, Ahmedabad, India, the samples were stored at − 65 ± 10 °C until completion of analysis. The serum samples were analysed using a standard, globally acceptable and validated bridging enzyme-linked immunosorbent assay (ELISA) method for anti-ranibizumab antibodies at Lambda Therapeutic Research Limited.

### Efficacy assessments

The efficacy assessments were done at baseline, Week 12 and end of the study (Week 24) using BCVA, central retinal thickness (CRT), and Visual Function Questionnaire (VFQ)-25 [23] parameters. The BCVA was assessed using the ETDRS visual acuity chart. The proportion of patients who lost fewer than 15 letters (approximately 3 lines) from baseline visual acuity at the end of the study, and mean increase in the BCVA in the study eye from baseline to the end of the study, were calculated. The central retinal thickness was measured by SD-OCT in the study eye and changes in the thickness of retina from baseline to the end of the study were calculated. The changes in VFQ-25 score from baseline to the end of the study were also measured.

### Sample size and statistical methods

Based on the requirement of the Indian apex drug regulator the ‘DCGI’ to generate the efficacy and safety data on Razumab in at least 100 patients, a total 126 patients were planned to be recruited into the study considering a drop out/withdrawal rate of ~ 20%. The safety variables were assessed using descriptive statistics such as number of observations, mean, standard deviation, median, and range for continuous variables, and percentages and frequency for categorical variables. The safety analysis was done on the safety population which was defined as patients who received at least one dose of biosimilar ranibizumab. The percentage of patients developing anti-ranibizumab antibodies during the 6 months of treatment along with the 95% CI was calculated in immunogenicity population, defined as the patients who received at least one dose of biosimilar ranibizumab in the study and had at least one pre-dose and one post-dose immunogenicity sample analysed.

The efficacy variables including change in BCVA, CRT and VFQ-25 were assessed using descriptive statistics and the p-value was calculated using paired *t*-test to compare changes from the baseline to 24 weeks. The proportion of patients who lost fewer than 15 letters was presented. The efficacy analysis was done on an intent-to-treat (ITT) and per protocol (PP) populations. The ITT population was defined as all patients who received at least one dose of study treatment and had at least one efficacy assessment data. The PP population was defined as patients who completed the study without major protocol deviations impacting efficacy evaluations. The last observation carried forward (LOCF) approach was used to compute the missing data. Statistical analyses were done using SAS® Version 9.4 (SAS Institute Inc., USA).

## Results

### Patient disposition

**Fig. 2:**
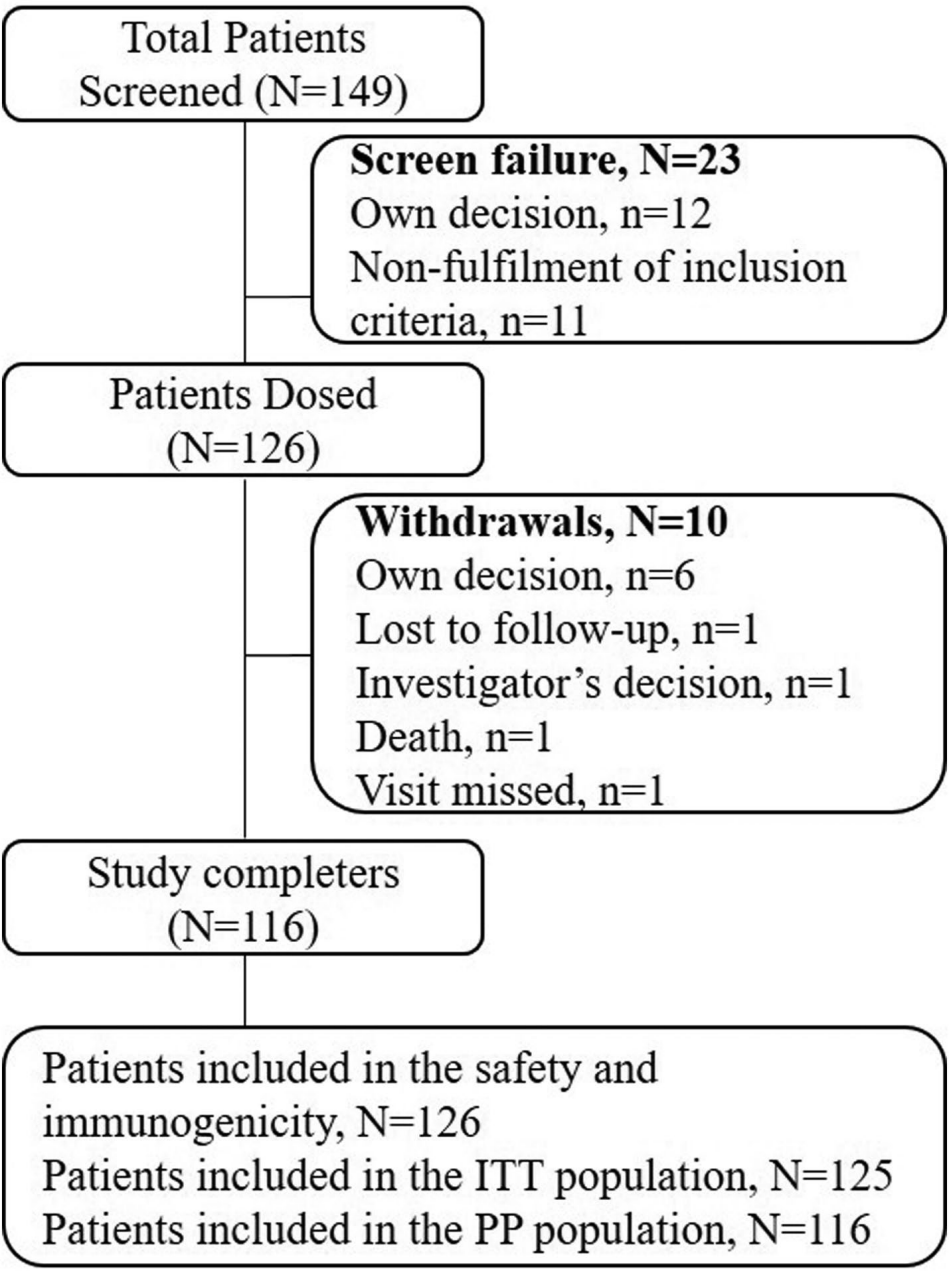
Patients disposition

A total of 149 patients were screened from 16 centres in India. Of which, 126 were enrolled and 23 were excluded as they did not meet the selection criteria (n = 11) or were not ready to provide consent for study participation (n = 12). All the 126 enrolled patients received biosimilar ranibizumab and were included in the safety and immunogenicity populations. The ITT population included 125 patients since one patient did not have any post-dose efficacy assessment due to deviation from the protocol procedure. Of the 126 patients, 116 (92.06%) completed the study and were included in the PP population; 10 (7.94%) patients did not complete the study since they did not return for follow-up (n = 1), died (n = 1), missed the visit (n = 1), investigator’s decision (n = 1) or withdrew consent (n = 6) (Fig. 2).

### Demographics and baseline characteristics

Of 126 patients, majority (72.22%, n = 91) were males (Table 1). All patients were Asians (Indians). Hypertension (35.71%) and diabetes (23.80%) were the most common comorbidities. For ITT and PP populations, the baseline mean ± SD BCVA were 44.0 ± 16.27 and 44.4 ± 16.38 letters, CRT were 384.8 ± 146.44 and 379.7 ± 141.57 µm, intraocular pressure (IOP) for left eye were 14.4 ± 3.31 and 14.6 ± 3.33 mm Hg, IOP for right eye were 14.2 ± 3.12 and 14.3 ± 3.13 mm Hg, and VFQ-25 score were 60.9 ± 14.36 and 60.4 ± 14.14, respectively.

**Table 1:** Baseline patients characteristics

Parameter (units)	Safety population (N = 126)	ITT population (N = 125)	PP population (N = 116)
Age (years), mean ± SD	68.75 ± 8.64	68.65 ± 8.61	68.69 ± 8.61
Height (cm), mean ± SD	159.91 ± 9.72	160.11 ± 9.50	159.97 ± 9.34
Weight (kg), mean ± SD	63.52 ± 9.90	63.60 ± 9.90	63.45 ± 9.54
BMI (kg/m^2^), mean ± SD	24.94 ± 4.16	24.91 ± 4.15	24.90 ± 4.11
Gender, n (%)			
Female	35 (27.78)	35 (28)	32 (27.59)
Male	91 (72.22)	90 (72)	84 (72.41)
Study eye, n (%)			
Left	51 (40.48)	50 (40)	46 (39.66)
Right	75 (59.52)	75 (60)	70 (60.34)

*BMI* body mass index, *ITT* intent-to-treat, *PP* per-protocol, *SD* standard deviation

### Biosimilar ranibizumab exposure

All 126 enrolled patients received at least one dose of biosimilar ranibizumab, of which 120 (95.24%) patients received all 6 doses of biosimilar ranibizumab (total 3 mg), 4 (3.17%) patients received 4 doses (total 2 mg) and 1 patient each received five doses (total 2.5 mg) and one dose (total 0.5 mg).

### Safety

Of 126 patients, 16 (12.7%) patients reported a total 19 AEs during the study (Table 2). A patient with history of hypertension and asthma and who had myocardial infarction (MI) 4 months before screening died following the 4^th^ dose of biosimilar ranibizumab due to another MI event which was unrelated to the study drug. Except for this patient, none discontinued the study due to an AE. There were no other SAEs reported in the study.

**Table 2:** Adverse events (safety population)

System organ class Preferred term	Biosimilar ranibizumab (N = 126) N (%) e
Total events	16 (12.70%) 19
Eye disorder	6 (4.76%) 6
Corneal edema	1 (0.79%) 1
Dry eye	1 (0.79%) 1
Eye pruritus	2 (1.59%) 2
Iridocyclitis	1 (0.79%) 1
Ocular hyperaemia	1 (0.79%) 1
General disorders and administration site conditions	6 (4.76%) 6
Death	1 (0.79%) 1
Pyrexia	5 (3.97%) 5
Infections and infestations	1 (0.79%) 1
Nasopharyngitis	1 (0.79%) 1
Investigations	4 (3.17%) 5
Raised blood pressure	1 (0.79%) 1
Increase in intraocular pressure	3 (2.38%) 4
Nervous system disorders	1 (0.79%) 1
Headache	1 (0.79%) 1

*N* Number of patients, *e* Number of events

Adverse events were categorized using MedDRA Version 20.1

Out of 19 AEs, only two AEs, iridocyclitis and increase in intraocular pressure, were considered related to the study drug. The mean ± SD IOP (mm Hg) decreased from baseline 14.5 ± 3.38 to 13.9 ± 2.74 for left eye, and from 14.2 ± 3.19 to 13.9 ± 2.97 for right eye at Week 24. Majority of the AEs were mild (n = 15), 3 AEs (corneal edema, iridocyclitis, increase in intraocular pressure) were moderate, and one AE (death) was severe. Except death, all other AEs resolved during the study. Out of 19 AEs, 10 AEs were ocular and were reported by 9 (7.14%) patients. The most common ocular AE was increase in intraocular pressure (4 AEs in 3 patients), followed by eye pruritus (2 AEs in 2 patients). The most common non-ocular event was pyrexia (5 AEs), reported in 5 (3.97%) patients. No event of hypersensitivity reaction to biosimilar ranibizumab was reported in the study.

### Immunogenicity

A total of 10 (7.94%; 95% CI 3.22%, 12.66%) patients were positive for anti-ranibizumab antibodies prior to dosing, and 9 (7.14%; 2.65%, 11.64%), 5 (3.97%; 0.56%, 7.38%), 6 (4.76%; 1.04; 8.48%), 5 (3.97%; 0.56%, 7.38%), 8 (6.35%; 2.09%, 10.61%), 9 (7.14%; 2.65%, 11.64%), 9 (7.14%; 2.65%, 11.64%), and 8 (6.35%; 2.09%, 10.61%) patients at Weeks 2, 4, 6, 8, 12, 16, 20 and 24, respectively, were positive for anti-ranibizumab antibodies post treatment with biosimilar ranibizumab.

### Efficacy

At the end of 24 weeks, visual acuity of treated eye improved in both ITT and PP populations as assessed by efficacy parameters such as the 15-letter loss for visual acuity, BCVA, CRT and VFQ-25 score.

**Fig. 3:**
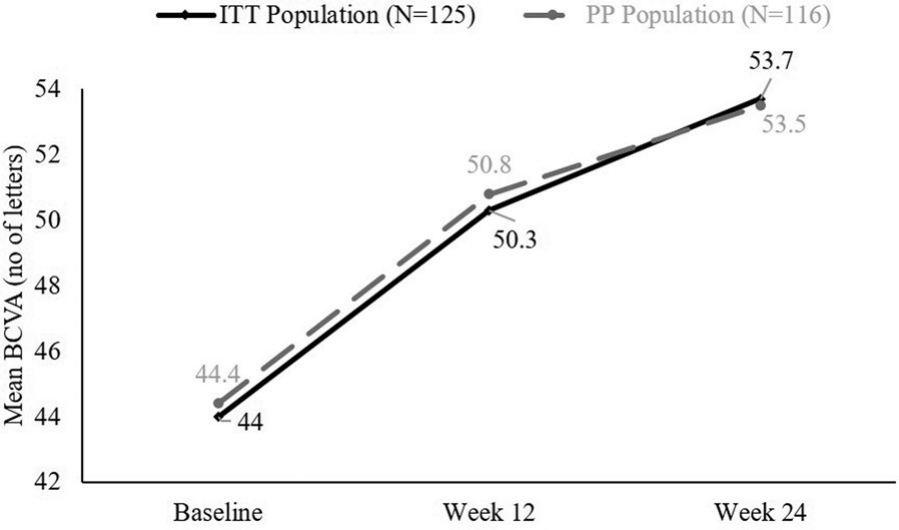
Improvements in BCVA from baseline to end of the study. *BCVA* best corrected visual acuity, *ITT* intent-to-treat population, *PP* per-protocol population

The proportion of patients who lost fewer than 15 letters from baseline visual acuity was 98.40% (95% CI 96.20%, 100.60%) patients at Week 12 and 97.60% (95% CI 94.92%, 100.28%) patients at Week 24 in the ITT population, and 98.28% (95% CI 95.91%, 100.64%) patients at Week 12 and 97.41% (95% CI 94.53%, 100.30%) patients at Week 24 in the PP population. The mean (SD) BCVA improved significantly from 44 (16.27) to 50.3 (17.37) letters at Week 12 (mean [SD] difference 6.3 [11.11] letters, p < 0.0001) and 53.7 (17.83) letters at Week 24 (mean [SD] difference 8.8 [13.61] letters, p < 0.0001) in the ITT population and from 44.4 (16.38) to 50.8 (17.17) letter at Week 12 (mean [SD] difference 6.5 [11.31] letters, p < 0.0001) and 53.5 (17.84) letters at Week 24 (mean [SD] difference 9.2 [13.85] letters, p < 0.0001) in the PP population (Fig. 3). The proportion of patients who showed improvement in the visual acuity by ≥ 15 letters from baseline to Weeks 12 and 24 were 23.20% and 31.20% for the ITT, and 24.14% and 32.76% for the PP populations, respectively.

**Fig. 4:**
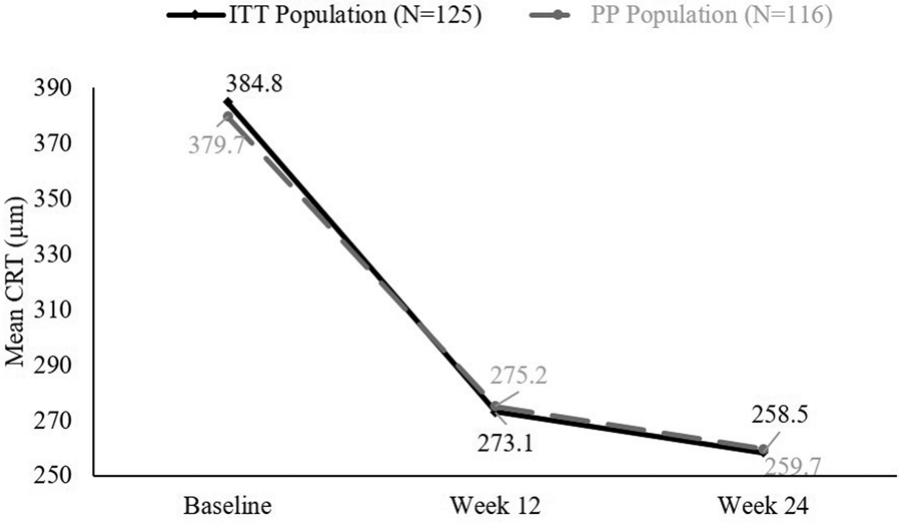
Improvements in CRT from baseline to end of the study. *CRT* central retinal thickness, *ITT* intent-to-treat population, *PP* per-protocol population

The mean (SD) CRT significantly decreased from 384.8 (146.44) µm to 273.1 (101.34) µm at Week 12 (mean [SD] difference 110.6 [130.65] µm, p < 0.0001) and 258.5 (74.77) µm at Week 24 (mean [SD] difference 125 [130.37] µm, p < 0.0001) in the ITT population and from 379.7 (141.57) µm to 275.2 (102.59) µm at Week 12 (mean [SD] difference 104.1 [126.73] µm, p < 0.0001) and 259.7 (74.00) µm (mean [SD] difference 119.3 [127.51] µm, p < 0.0001) in the PP population (Fig. 4). The VFQ-25 scores also significantly improved from baseline to the end of the study (Table 3).

**Table 3:** Visual function questionnaire-25 score

VFQ-25 Score	ITT population (N = 125)	PP population (N = 116)
Baseline, mean ± SD	60.9 ± 14.36	60.4 ± 14.14
Week 12, mean ± SD	66.7 ± 12.65	66.5 ± 12.50
Mean ± SD change from baseline to Week 12	5.8 ± 10.78 [p < 0.001]	6.1 ± 10.96 [p < 0.001]
Week 24, mean ± SD	69.5 ± 12.46	69.5 ± 12.35
Mean ± SD change from baseline to Week 24	8.5 ± 13.04 [p < 0.001]	9.2 ± 13.33 [p < 0.001]

*ITT* intent-to-treat population, *PP* per-protocol population

## Discussion

The ASSET was a phase 4, single arm post-marketing, prospective study conducted to evaluate the safety and efficacy of the world’s first biosimilar ranibizumab, Razumab™, in patients with wet AMD. The biosimilar ranibizumab was well-tolerated in patients with wet AMD and showed a similar safety profile as that of the innovator ranibizumab reported in the literature [24]. The biosimilar ranibizumab was found efficacious in improving the overall wet AMD disease condition. The efficacy and safety of biosimilar ranibizumab has also been demonstrated in the previous real-world retrospective RE-ENACT and RE-ENACT 2 studies in patients with several macular disorders including wet AMD [18, 21].

In this study, we enrolled 126 patients of either gender with age ≥ 50 years who had BCVA of 20/40 to 20/320 using ETDRS chart and who had active sub-foveal CNV in the study eye. Active CNV and age ≥ 50 years are the major risk factors of wet-AMD patients leading to severe vision loss or blindness in the patients [25]. The study population was selected based on the innovator’s ANCHOR, MARINA, HARBOR, PIER, and SUSTAIN studies in wet-AMD patient population [26–30]. The ANCHOR and MARINA studies have demonstrated that monthly ranibizumab regimen provides better benefits compared to other regimens with less frequent dosing as seen in HARBOR, PIER, SUSTAIN and IVAN studies [26–31]. Similarly, in our cohort, patients were administered 0.5 mg monthly regimen of intravitreal biosimilar ranibizumab.

In our study, patients were dosed for 6 months to assess the safety and efficacy of biosimilar ranibizumab, which was chosen considering improvement in visual acuity of wet AMD patients after receiving monthly ranibizumab in ANCHOR, MARINA, EXITE, SUSTAIN, and HARBOR studies [26–28, 30, 32]. These studies showed significant improvement in visual acuity by three months and patients could sustain the benefit till the end of the treatment period of 12 or 24 months. Therefore, we chose to assess efficacy of biosimilar ranibizumab till 6 months (total 6 doses). This was also supported by ProNTO and LUMIERE studies where patients received an average of 5 or 6 doses [33, 34]. We assessed the safety of biosimilar ranibizumab using standard eye examinations including fundus fluorescein angiography, slit-lamp examination, indirect ophthalmoscopy, and laboratory assessments [35, 36]. The efficacy of biosimilar ranibizumab was assessed using sensitive endpoints such as change in BCVA using ETDRS chart, change in CRT measured by SD-OCT, and changes in VFQ-25 which assessed vision-related activities during day to day life [37].

Of the 19 AEs reported in 16/126 (12.7%) patients, 10 were ocular AEs reported in 9 (7.14%) patients; increase in intraocular pressure was the most common AE. Of 4 AEs of increase in intraocular pressure, only one was considered related to the study drug. Increase in intraocular pressure have been noted both pre- and post-intravitreal injections [38]. The mean changes in IOP at the end of study were minimal (< 1 mm Hg) and not significant in this study, similar to that reported in previous studies of biosimilar ranibizumab [17–19].

Iridocyclitis, corneal edema, ocular hyperaemia, and dry eye were observed in < 1% of patients after biosimilar ranibizumab treatment. Similar incidence rates for these AEs were observed in the innovator ranibizumab postmarketing surveillance studies [7, 39]. The common (> 10%) ocular AEs reported for ranibizumab are conjunctival haemorrhage, eye pain, vitreous detachment or floaters, increase in intraocular pressure, intraocular inflammation, visual disturbance, eye irritation, increased lacrimation, blepharitis, dry eye, ocular hyperaemia and eye pruritus [38].

In the current study, hypertension and nasopharyngitis were reported in one patient each. Incidence rates of hypertension, nasopharyngitis and headache ranged from 3 to 9%, while incidence rates of thromboembolic events ranged from 0.8 to 5.6% with innovator ranibizumab as reported in literature [26, 27, 30, 32, 40, 41]. Non-ocular AEs associated with systemic VEGF inhibition, such as arterial thromboembolic events, hypertension, proteinuria, and non-ocular haemorrhage are of particular interest [9, 12].

Of 126 patients, only one patient discontinued the study due to an AE, i.e. death due to MI. This AE was considered unrelated to the study drug. Important ocular SAEs such as endophthalmitis and vitreous haemorrhage, and non-ocular SAEs such as thromboembolic events, which were reported in some large clinical studies of innovator ranibizumab, were not reported in our study [26, 27, 30, 42, 43]. However, it is noteworthy that many interventional and observational postmarketing studies have also not reported any case of endophthalmitis [30, 32, 39]. In our study, anti-ranibizumab antibodies were seen in 7.94% patients before treatment and in 7.14% patients after biosimilar ranibizumab treatment, which is comparable to the pre-treatment immunoreactivity incidence of 0–5% and 1–9% after 6 to 24 months of innovator ranibizumab treatment [38]. In a study by Nicolas et al. the incidence of immunogenicity was 17.1% with innovator ranibizumab [44]. Moreover, there was no increased incidence of immunogenicity with the increase in the number of biosimilar ranibizumab injections administered in our study.

Patients with wet AMD lose visual acuity by 2 to 3 lines per year if the patient remains untreated [27, 45]. Biosimilar ranibizumab treatment showed benefit in visual acuity during the 24 weeks of treatment. At the end of the study (Week 24) for ITT and PP populations, 97.60% and 97.41% patients lost fewer than 15 letters, respectively, and visual acuity improved by 15 letters for 31.20% and 32.76% patients, respectively, from baseline to the end of the study. In ANCHOR and MARINA studies, 96.4% and 94.6% patients lost fewer than 15 letters respectively and visual acuity increased by 15 letters for 40.3% and 33.8% patients respectively after receiving a total of 12 doses of ranibizumab [26, 27]. The improvement in BCVA after 6 months of biosimilar ranibizumab treatment was 9.2 letters which was in line with 10.6 letters reported in the ANCHOR and 6.5 letters reported in the MARINA studies [26, 27]. The improvement in CRT (119.3 µm) observed in this study was also comparable with other studies of ranibizumab in patients with wet AMD [30].

The study limitations included the unavailability of details pertaining to the patterns, size and type of CNV (classic/occult/mixed). Also, the details of the patients who had received previous treatments before the timelines as mentioned in the exclusion criteria, were not captured, and hence, the data on ‘treatment naïve’ versus ‘previously treated patients’ could not be provided.

## Conclusions

Overall, this prospective, postmarketing ASSET study of the world’s first biosimilar ranibizumab, Razumab™, in patients with wet AMD over 6 months showed similar safety and efficacy profiles to the evidence reported with innovator ranibizumab. Biosimilar ranibizumab was well-tolerated without any new safety concerns. Biosimilar ranibizumab also showed similar improvement in patients with wet AMD for 15 letter loss assessed by visual acuity, BCVA and retinal thickness, as reported with innovator ranibizumab. Long-term studies with a larger patient population may elicit better results.

## Supplementary Information

**Additional file 1: Appendix S1.** Safety Assessment Questionnaire

## Data Availability

The datasets used and/or analysed during the current study are available from the corresponding author on reasonable request.

## Abbreviations

AE: Adverse event
AMD: Age-related macular degeneration
ASSET: rAnibizumab bioSimilar Safety Efficacy postmarkeTing
BCVA: Best corrected visual acuity
CNV: Choroidal neovascularization
CRT: Central retinal thickness
DCGI: Drugs Controller General of India
DME: Diabetic macular edema
ELISA: Enzyme-linked immunosorbent assay
ETDRS: Early Treatment Diabetic Retinopathy Study
FFA: Fundus fluorescein angiography
IOP: Intraocular pressure
ITT: Intent-to-treat
LOCF: Last observation carried forward
MedDRA: Medical Dictionary for Regulatory Activities
MI: Myocardial infarction
PP: Per protocol
RVO: Retinal vein occlusion
SAE: Serious adverse event
SD: Standard deviation
SD-OCT: Spectral domain-optical coherence tomography
VEGF: Vascular endothelial growth factor
VFQ: Visual Function Questionnaire
