# Supplementary figures and images for: Safety and efficacy of Razumab™ (world’s first biosimilar ranibizumab) in wet age-related macular degeneration: a post-marketing, prospective ASSET study
Source: Int J Retina Vitreous. 2021 Mar 24;7:24. doi: 10.1186/s40942-021-00293-w (PMC7992797; doi:10.1186/s40942-021-00293-w)

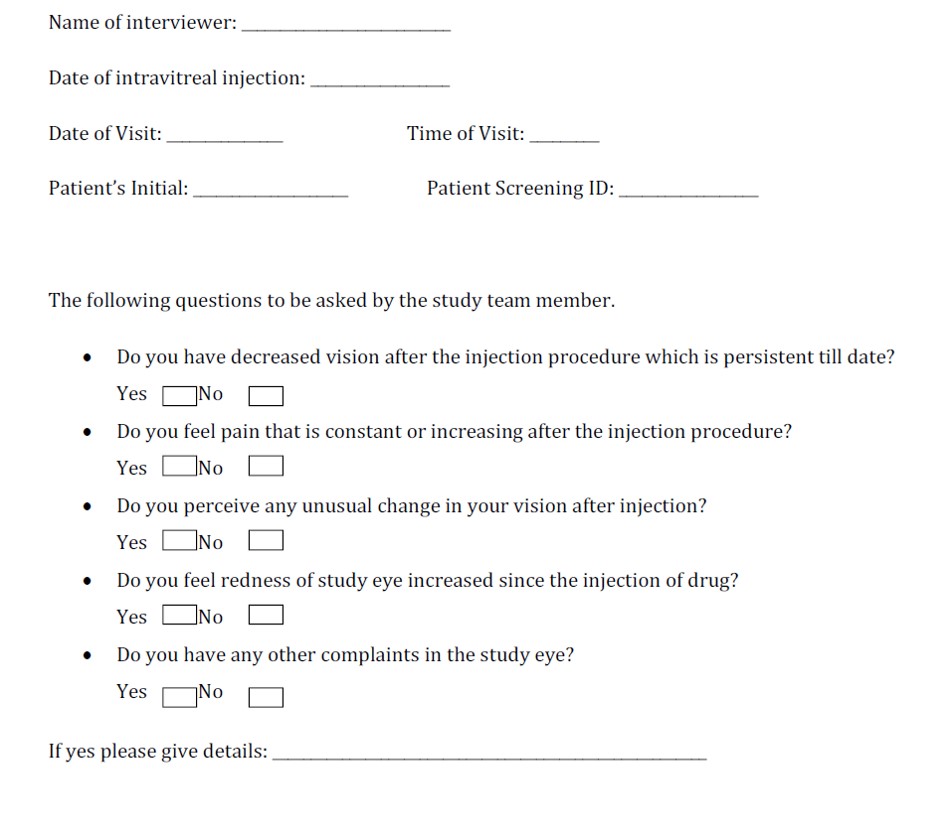

Supplement: Supplementary file 1 — Additional file 1: Appendix S1. Safety Assessment Questionnaire [file 40942_2021_293_MOESM1_ESM.jpg]
